# Editorial: Exploring the subcellular proteome and its post-translational modifications

**DOI:** 10.3389/fmolb.2022.994961

**Published:** 2022-08-10

**Authors:** Shen Zhang, Hao Jiang, Qi Wu

**Affiliations:** ^1^ Clinical Research Center for Reproduction and Genetics in Hunan Province, Reproductive and Genetic Hospital of CITIC-XIANGYA, Changsha, China; ^2^ School of Life Sciences, University of Dundee, Dundee, United Kingdom; ^3^ Department of Biomedicine, Aarhus University, Aarhus, Denmark

**Keywords:** subcellular, proteomics, proteome, post-translational modification, cell compartment

Study of the subcellular proteome and its post-translational modifications (PTMs) is essential to the understanding of the dynamics of protein regulation in various parts and/or compartments of cells (Christopher et al., 2021). In this Research Topic, we published three primary research articles focusing not only on the understanding of regulatory roles of the subcellular proteome and its PTMs, but also its development of advanced technologies and applications. In addition, a mini-review was published to summarize and discuss the technological strategies in compartmentalized proteome studies.

The first primary study is on the apoptosis of vascular smooth muscle cells (VSMCs). Apoptosis of vascular smooth muscle cells (VSMCs) is closely related to the pathogenesis of cardiovascular disease, and oxidative stress is an important cause for the death of VSMCs. Therefore, inhibiting VSMCs apoptosis is an effective preventive strategy in slowing down the development of cardiovascular disease. Effective antioxidant is imperatively needed to attenuate the oxidative stress-induced VSMCs apoptosis. Ginger is a common spices and has many bioactive compounds with pharmacological activities. In the study by Liu et al., they found OXR1 (oxidation resistance protein 1), a crucial participant that can respond to oxidative stress, could modulate the expression of p53, a key regulator of apoptosis. In addition, 6-shogaol (6S), a major biologically active compound in ginger, could effectively attenuate cell death by preventing the up-regulation of OXR1-p53 axis. Furthermore, quantitative proteomic analysis revealed that enhanced assembly of SKP1-CUL1-F-box protein ubiquitin ligase complexes might facilitate the degradation of p53 mediated by OXR1.

The second study is on acetylation-dependent protein-protein interactions. The acetylation of lysine residues (Kac) on histones can act as anchor points for bromodomain-containing adapter proteins, promoting interactions of cellular networks to regulate gene transcription. In the study by Loehr et al., they established a cell model in which cellular Kac level is dependent on extracellular acetate level. They used gene editing to knock out ATP citrate lyase, disrupting citrate-to-acetyl-CoA conversion in the cytoplasm and nucleus. The essence of ATP citrate lyase can be overcome through acetate supplementation, enabling acetyl-CoA production via the acetyl-coenzyme A synthetase pathway. As a result, the Kac level was sensitive to the extracellular acetate level. The results demonstrated the capacity of this model to modulate the Kac expression. Using this model, both global and specific reorganizations of protein interaction networks in response to Kac changes were characterized. In conclusion, this work developed an effective, fast and scalable cell model that will benefit the investigation of acetylation-dependent protein-protein interactions.

The third study is on SUMOylation (Small Ubiquitin-like MOdifier) of the kidney distal convoluted tubule (DCT) cells. SUMOylation on lysine residue is a reversible process that regulate transcription and protein stability. In the kidney, SUMOylation seems to be important for the cellular response to aldosterone. Therefore, in the study by Aroankins et al., they profiled the SUMOylation landscape of a modified mouse kidney DCT cell line (mpkDCT) as a starting point to understand the SUMOylation events in this cell type. SUMOylation of one particular transporter, the renal hydrogen-coupled oligopeptide and drug co-transporter (Pept2), at one particular site (K139), was found to be highly regulated by aldosterone. Pept2 expression increased up to four-fold post-transcriptionally when mpkDCT cell lines expressing wild-type Pept2 or mutant K139R-Pept2 were treated with aldosterone. Pept2 abundance decreased in the apical membrane of mpkDCT cells after aldosterone stimulation in wild-type expressing cells, but not in K139R-Pept2 expressing cells. All these suggest that SUMOylation plays an important role in the physiological regulation of Pept2 trafficking by aldosterone.

In the technological mini-review by Dionne et al., the authors summarized and discussed the progress and limitations of historically and currently used strategies to investigate compartmentalized proteome. The unique advantages and the intrinsic caveats of each proximity-dependent biotinylation (PDB) strategy were also elaborated to provide a deeper understanding of the conceptual and applicable areas of these strategies. Furthermore, the potential of PDB to study dynamic and spatial PTMs were highlighted, pointing out the future directions of PDB in characterizing the roles of PTMs in spatial proteome regulation.

We believe that the broad range of contents covered in this Research Topic provides a forum for investigating the subcellular proteome and its PTMs, and we hope that further technological advances in this field could be the driving force for deeper biological insights.
